# *In silico* CD4 + T-cell multiepitope prediction and HLA distribution analysis for Marburg Virus—A strategy for vaccine designing

**DOI:** 10.1016/j.jksus.2021.101751

**Published:** 2021-12-06

**Authors:** Anupam Dhasmana, Swati Dhasmana, Ahmad Alsulimani, Sudhir Kotnala, Vivek Kumar Kashyap, Shafiul Haque, Meena Jaggi, Murali M. Yallapu, Subhash C. Chauhan

**Affiliations:** a Department of Immunology and Microbiology, School of Medicine, University of Texas Rio Grande Valley, Edinburg, TX, USA; b Department of Biosciences, Himalayan Institute of Medical Sciences, Swami Rama Himalayan University, Dehradun, India; c Medical Laboratory Technology Department, College of Applied Medical Sciences, Jazan University, Jazan, Saudi Arabia; d Research and Scientific Studies Unit, College of Nursing and Allied Health Sciences, Jazan University, Jazan, Saudi Arabia; e Bursa Uludağ University, Faculty of Medicine, Görükle Campus, 16059 Nilüfer, Bursa, Turkey

**Keywords:** Marburg Virus, Peptide based vaccine, CD4+ T Cell, Non-mutagenic, Antigenic, Non-toxic and High world population coverage

## Abstract

Marburg, a RNA virus (MRV), is responsible for causing hemorrhagic fever that affects humans and non-human primates. World Health Organization (WHO), National Institutes of Health (NIH) and Centre of Disease Control and Prevention (CDC) considered this as an extremely dangerous virus, thus categorised as risk group 4, category A priority pathogen and category "A” bioterrorism agent, respectively. Despite of all these alarming concerns, no prophylaxis arrangements are available against this virus till date. In fact, the construction of immunogenic vaccine candidates by traditional molecular immunology methods is time consuming and very expensive. Considering these concerns, herein, we have designed CD4 + T Cell multiepitopes against MRV using *in silico* approach. The pin-point criteria of the screening and selection of potential epitopes are, non-mutagenic, antigenic, large HLAs coverage, non-toxic and high world population coverage. This kind of methodology and investigations can precisely reduce the expenditure and valuable time for experimental planning in development of vaccines in laboratories. In current scenario, researchers are frequently using *in silico* approaches to speed up their vaccine-based lab studies. The computational studies are highly valuable for the screening of large epitope dataset into smaller one prior to *in vitro* and *in vivo* confirmatory analyses.

## 1. Introduction

Marburg virus (MRV) is genus of Filoviridae family. MRV is negative stranded & non-segmented RNA virus, that is responsible for severe hemorrhagic fever, known as marburg hemorrhagic fever (MHF) in both humans and non-human primates. The MRV infection has approximately 23 to 100% fatality and lethality rates in humans and non-human primates (Mehedi et al., 2011). The systemic viral replication of MRV interferes with immune and inflammatory activities, the consequence of which are serious pathological features in patients, like hemorrhages, edema, coagulation imbalance, multiple-organ failure and shock, often resulting in death (Bente et al., 2009). Before the discovery of Ebola in 1967, the first MRV infection was observed in Germany and Serbia (Mehedi et al., 2011) followed by Zimbabwe/South Africa in 1975 (Conrad et al., 1978) Angola in 2004 (Towner et al., 2006). According to the report of CDC 2014, the recent outbreak was in Uganda from 2007 to 2014. Few studies demonstrated that MRV is highly infectious and very stable in experimental aerosol exposure (Alves et al., 2010), which raises the concern that MRV may be very suitable to be used as biological weapon (US Centres for Disease Control and Prevention/"Bioterrorism Agents/Diseases” report). Despite of all, in current scenario no treatment is available against MRV infection (Cross et al., 2018). The main enviable feature for any vaccine candidate is that the molecule should activate cell-mediated (T-Cell) and humoral (B-Cell) immune response followed by memory cell formation. CD4 + T-cells activation is mandatory for a competent humoral immune response for the induction of Immunoglobulin-G and memory B cells. CD4 + T-cells primarily recognize antigen peptides by CD4 co-receptor and only recognize the major histocompatibility complex (MHC) II protein on antigen-presenting cells; then memory B cells make a repository of infected virus for the farther prophylaxis arrangement (Clem, 2011). *Filoviridae* family virus consists of 7 structural proteins, among all, highly glycosylated (N- and O-linked glycans) envelope glycoproteins (*Env* GPs) are present over the cell surface. Host proteases like furin is responsible for the proteolysis of GP, resulting in two subunits, GP1 and GP2, linked by a disulfide bond (Volchkov et al., 2000). These GPs mediate and lead the viral entry into host cells (Takada et al., 1997),(Wool-Lewis and Bates, 1998), thus GPs are considered to be the ideal target for neutralizing antibodies against filoviruses. In current era as the electronic support increases in the life sciences, computational based approach provides access to researchers to deal with huge number of genome and proteome data of virus. Immunogenic, non-toxic and peptide-based vaccines would prove to be a good alternative treatment option for the management of MRV infection. In this article, we have focused on the special epitopes candidates those have non mutagenic tendency, which were thoroughly screened via protein variability server. We have identified various small fragments of *Env* GP those don’t have the mutation hot spot, which will lead the less chance of viral resistance. In current COVID scenario scientists have encountered with the toxicity issue of vaccines so here we have seriously focused on the non-toxic and highly immunogenic multi-epitopes from *Env* GP proteins of MRV virus. The recognition of world-wide HLA coverage analysis of CD4 + T-cell epitopes in *Env* GP protein was carried out by using artificial neural network algorithm (ANN) implemented in IEDB and NetMHCIIPan Server. This article, is clearly depicted that the focus of this article is to provide a rapid, cost effective and efficient vaccine candidate and process by using epitopes of viral proteins. This strategy was designed to keep in the mind of current COVID situation where we were looking safe and effective vaccine. Since a very recent statement of WHO, also claimed the inflammable problem of marburg virus as a "scary & deadly disease”. (https://www.express.co.uk/news/science/1474916/marburg-virus-news-disease-scary-deadly-world-health-organisation-africa-spt)

## 2. Materials and methods

### 2.1. Sequence retrieval and multiple sequence alignment (MSA) of retrieved proteins

681 residue long amino acid sequences of viral *Env* GP from 37 different strains of MRV, that are involved in the host cell binding and fusion activity, were retrieved from UniProtKB Database (www.uniprot.org). The retrieved sequences were further subjected to multiple sequence alignment using CLUSTAL Omega, to spot the non-mutated and highly immunogenic amino acid sequences for the assessment and predictions of effective epitope.

### 2.2. Protein variability analysis of retrieved sequences

Protein Variability Server/PVS (http://imed.med.ucm.es/PVS/) was used to identify the variable or high mutational rate amino acid in the particular protein sequences, because one virus has several strains worldwide and they differ with each other’s on the basis of highly mutated amino acid, which is the natural tendency of viruses to show high mutational rates. This tendency is the reason of failure of most of the vaccines. (Garcia-Boronat et al., 2008)

### 2.3. Immunogenicity-antigenicity prediction of the viral protein

The VaxiJen V2.0 server (http://www.ddg-pharmfac.net/vaxijen/VaxiJen/VaxiJen.html) was used for the assessment of immunogenicity-antigenicity of the selected protein sequences from PVS. This server runs on Auto Cross Covariance (ACC) algorithm that predicts protective and tumor antigens and subunit vaccines with the accuracy level of up to 89 %. (Doytchinova and Flower, 2007), (Janahi et al., 2017)

### 2.4. CD4 + epitope prediction

The *Env* GP protein sequence was investigated for the screening of the probable leading T-cell CD4 + epitopes using bioinformatics tool NetMHCIIpan server (Nielsen et al., 2008), which is one of the most accurate prediction servers currently available based on ANN. NetMHCIIpan server have huge pool of more than 5000 HLAs (DQ: 2912, DP: 2247, DRB4: 06, DRB1: 15, DRB3: 29, DRB5:15). The predictions output, showing binding affinity of each epitopic from core sequences with every known HLA allele. The window of peptide length was set to be 15 for HLA-II, respectively as mentioned in earlier publications (Janahi et al., 2017),(Bano et al., 2018). The epitopes were predicted on the basis of lowest percentile rank and high binding affinity.

### 2.5. Identification of non-toxic region of selected epitopes

The final selected epitopes were checked for the conserved regions and further subjected to ToxinPred severe (Gupta et al., 2013) for the segregation of toxic or nontoxic peptides. Support Vector Machine (SVM) and Quantitative Matrix based algorithm were used to generate quantitative matrix on the basis of probability or frequency of amino acid at a particular location.

### 2.6. Population coverage analysis

**Fig. 1.**
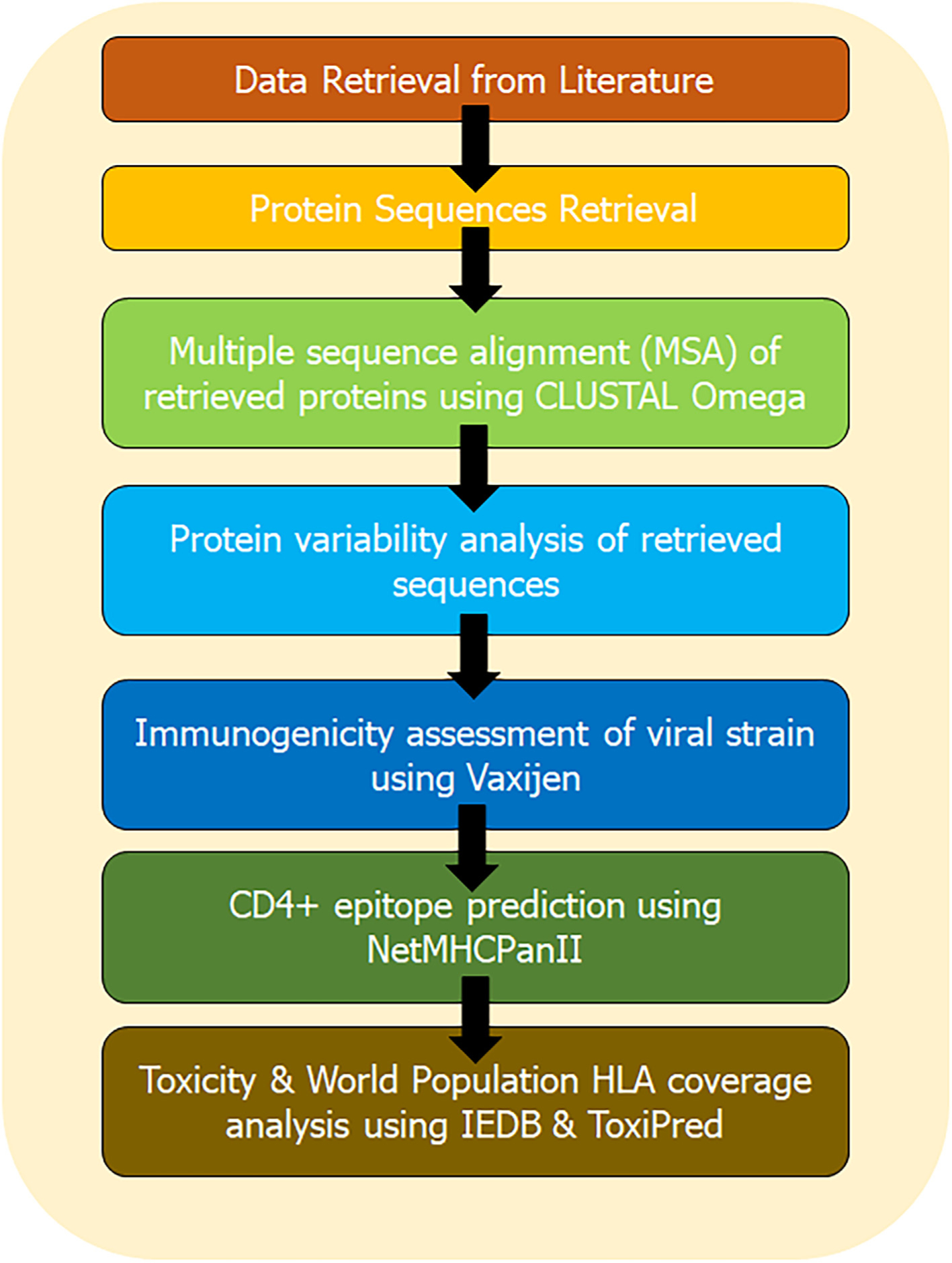
The schematic representation of the entire methodology.

The population coverage rate of the final selected epitopes was calculated by using the IEDB population coverage tool (http://tools.immuneepitope.org/tools/population/iedb_input) (Bui et al., 2006). The predicted epitopes with their all-binding HLA alleles for the worldwide distribution were tabulated. IEDB server extract all allele genotypic frequencies related data from Allele Frequency database, which comprises with allele frequencies form huge population set of 115 countries and 21 different ethnicities grouped into 16 different geographical areas. The schematic representation of the entire methodology of *in silico* CD4 + T-cell epitope prediction and HLA distribution of MRV is mentioned in Fig. 1.

### 2.7. IFN-gamma inducing capacity and physiochemical prediction

IFN-gamma inducing capacity predictions of all 11 epitopes were predicted by IFNepitope server (http://crdd.osdd.net/raghava/ifnepitope/index.php). In this segment all final 11 epitopes were assessed by using two categories INF-G vs non-INF-G epitopes and INF-G vs other cytokines. Followed by physiochemical properties of finally selected epitopes by using various servers like https://pepcalc.com/; https://web.expasy.org/protparam/ & https://www.biosyn.com/peptidepropertycalculator/peptidepropertycalculator.aspx.

## 3. Results

### 3.1. Retrieval of Env glycoprotein sequences of MRV and multiple sequences alignment (MSA)

**Fig. 2.**
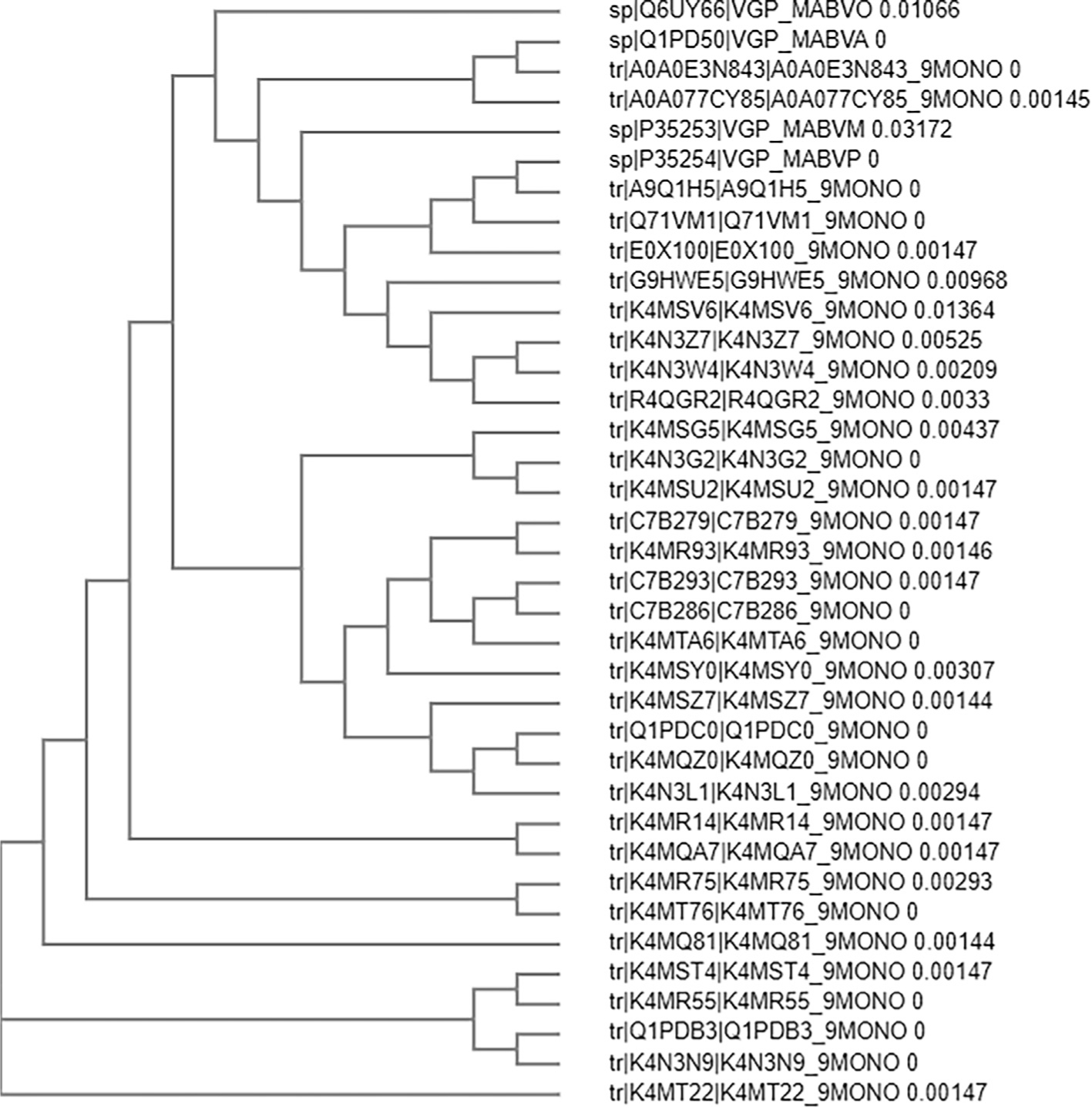
Phylogenetic relationship between all 37 *Env* proteins of all strains of Marburg virus.

**Fig. 3.**
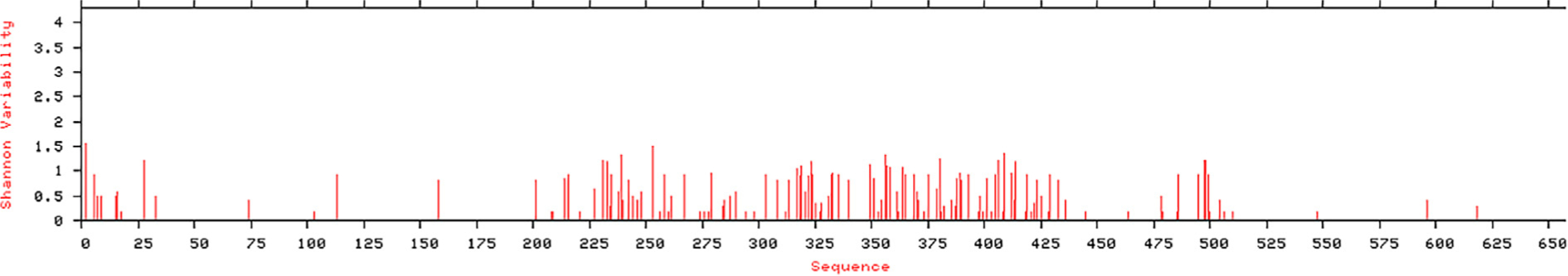
Protein Variability Plot of *Env* proteins of all 37 Sequences of Marburg Virus (MRV).

All 37 *Env* glycoprotein sequences of different strains of MRV (mentioned in Supplementary Data Set part 1) with more than 89% of similarity, were retrieved from uniprot database. CLUSTAL Omega was used for the identification of evolutionary relationship (as shown in Fig. 2) and percent of similarity between all 37 protein sequences of *Env* protein of **MRV** (mentioned in Supplementary Data Set part 2). Q6UY66|VGP_MABVO was considered as reference sequence and its comparative sequence similarity coverage with other proteins more than 89%. On the basis of similarity coverage, authors obtained the information about the variation in the sequences that may lead to hot points of the virus mutations (Fig. 3). Considering this, Protein Variability Server (PVS) was used to select the non-variable fragments of the viral *Env* proteins, which were used for the selection and identification of the most effective and immunogenic epitopes. Total ten fragments were obtained (Table 1) by PVS study. Often, only eight fragments were selected for the identification of epitopes because fragment no. 03 (PEIKPTSTPTDAT, 13 amino acids from 240 to 252) and 06 (NLSTLS, 06 amino acids from 350 to 354) have very lesser numbers of amino acids than the selection windows length for HLA-II peptide which was 15.

### 3.2. Antigenicity prediction of the viral protein fragments

The VaxiJen V2.0 online server was used for identification of antigenicity of the viral envelope (*Env*) protein fragments of different strains of MRV, by keeping the threshold at 0.4 (Table 1). The results obtained suggest that the viral protein fragments were probable antigens with a score of 0.6525 (Set 1, TTCFFISLILIQGIKTLPILEIASN from 3 to 27 position), 0.4850 (Set 2, QPQNVDSVCSGTLQKTEDVHLMGFTLSGQKVADSPLEASKRWAFRTGV PPKNVEYTEGEEAKTCYNSVTDPSGKSLLLDPPTNVRDYPKCKTIHHIQG QNPHAQGIALHLWGAFFLYDRIASTTMYRGKVFTEGNIAAMIVNKTVH KMIFSRQGQGYRHMNLTSTNKYWTSSNGTQTNDTGCFGTLQEYNSTKN QTCAPSK, from 29 to 230), 0.4729 (Set 4, LNTTNPNSDDEDLTTSG SGSGEQEPYTTSDAVTKQGLSSTMPPTPSPQPGTPQQGGNNTNHSQ, from 254 to 316), 0.4109 (Set 5, NTNTTAQPPMPSHNTTTISTNNTSK from 324 to 348), 0.6994 (Set 7, NTQSMATENEKTSAP, from 365 to 379), 0.4557 (Set 8, KTTLPPTESPTTEKSTNNTKSPTTM, from 381 to 405), 0.6654 (Set9, SPSSTPNSTTQHLIYFRRKRSILWREGDMFPFLD GLINAPIDFDPVPNTKTIFDESSSSGASAEEDQHASSNISLTLSYLP, from 415 to 496) and 0.4865 (Set 10, SENTAYSGENENDCDAELRIWSVQ EDDLAAGLSWIPFFGPGIEGLYTAGLIKNQNNLVCRLRRLANQTAK SLEL LLRVTTEERTFSLINRHAIDFLLTRWGGTCKVLGPDCCIGIEDLSRNISEQID QIKKDEQKEGTGWGLGGKWWTSDWGVLTNLGILLLLSIAVLIALSCICRI FTKYIG, from 498 to 680).

### 3.3. HLAs distribution analysis, antigenicity and toxicity profiling

NetMHCIIpan server was used for the identification and screening of putative CD4 + T-cell core epitope sequences among the 08 protein fragments of *Env* protein. Total 29 putative T-cell epitopes were extracted from *Env* protein, as shown in Table 2. ILIQGIKTLPILEIA was the best epitope which has highest HLA coverage (1034 no. of HLA covers), extracted, and screened from *Env* protein fragments. Of all 29 epitopes, 14 epitopes were identified as probable antigens. ToxinPred server was used for the toxicity profiling of all peptides (whether these peptides were toxic or non-toxic). The resultant of this profiling was that all peptides were found in non-toxic category.

### 3.4. Population coverage analysis

**Fig. 4.**
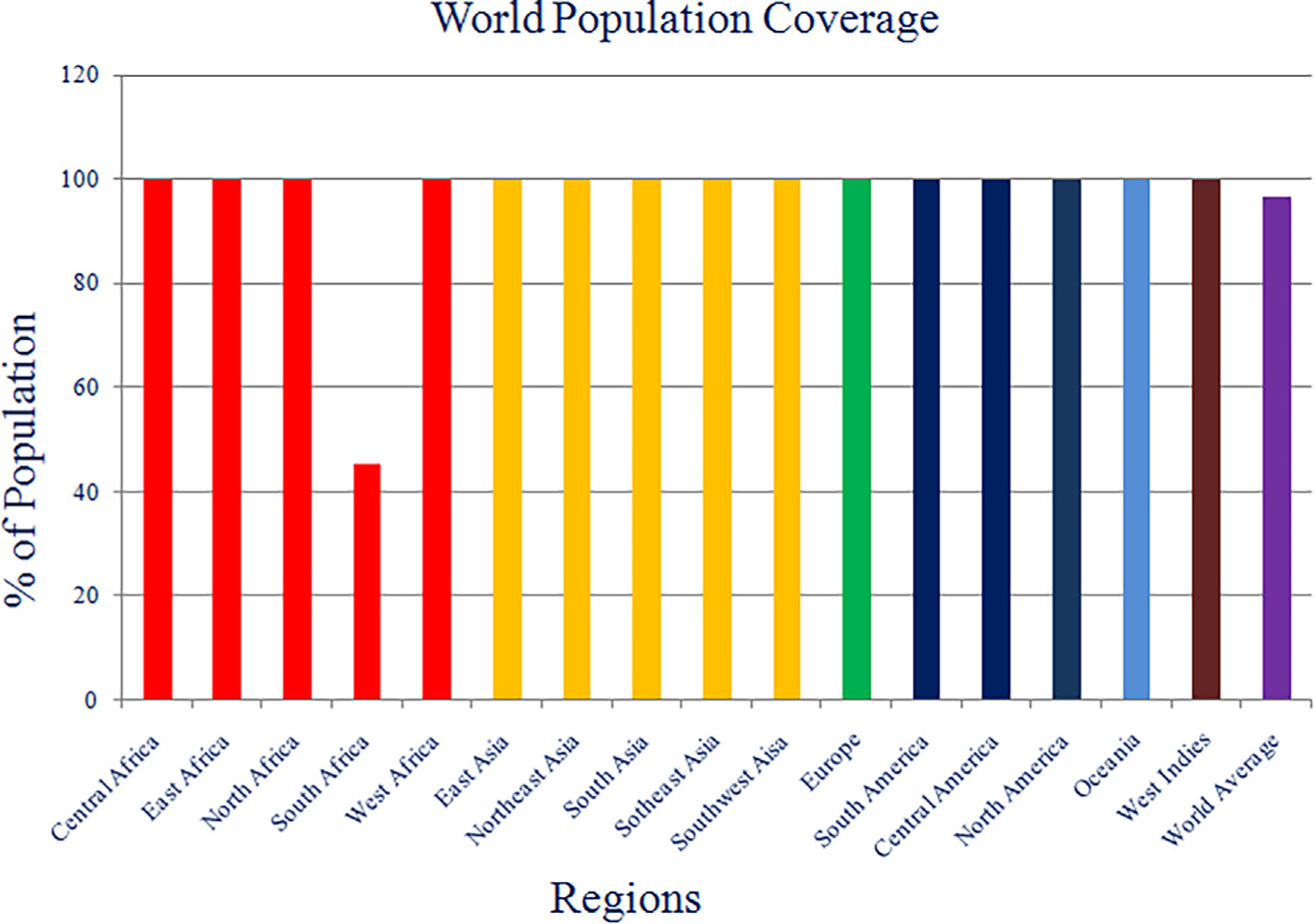
Bar-diagram representation of population coverage of final 11 epitopes against different regions of globe.

IEDB population coverage server was used for the identification of population coverage of our screened non-mutagenic, antigenic and non-toxic peptides. This analysis indicates that 11 peptides (ILIQGIKTLPILEIA, LILIQGIKTLPILEI, VFTEGNIAAMIVNKT, AFFLYDRIASTTMYR, FTEGNIAAMIVNKTV, QHLIYFRRKRSILWR, LIQGIKTLPILEIAS, SKRWAFRTGVPPKNV, WGAFFLYDRIASTTM, FDESSSSGASAEEDQ and TAGLIKNQNNLVCRL) show highest HLA coverage. Each region has 100% population coverage except South Africa, which makes an average of 96.79% of world population coverage, as mentioned in Fig. 4.

### 3.5. IFN-gamma inducing capacity and physiochemical prediction

IFN-gamma induction potential has been screened for all 11 epitopes by using two categories, IFN-G vs non-IFN-G and INF-G vs other cytokines. In first of category (INF-G vs non-INF-G) 05 out 11 epitopes ILIQGIKTLPILEIA, LILIQG IKTLP ILEI, FFLYDRIASTTMYR, QHLIYFRRKRSILWR and LIQGIKTLPILEIAS were qualified to inducing INF-gamma potential. Meanwhile we have again cross checked all 11 epitopes in the second category (INF-G vs other cytokines), and we found that all 11 epitopes were qualified and had more potential to induce the IFN-gamma than other cytokines (Table 3). The physiochemical parameters of 3 epitopes QHLIYFRRKRSILWR, SKRWAFRTGVPPKNV and FDESSSSGASAEEDQ have hydrophilic nature; the rest of them were fall in hydrophobic in nature. AFFLYDRIASTTMYR, LIQGIKTLPILEIAS and WGAFFLYDRIASTTM epitopes were shown thermodynamically stable in biological systems, the rest of them were unstable. ILIQGIKTLPILEIA, LILIQGIKTLPILEI and LIQGIKTLPILEIAS were found to have good protein-binding potential according to Boman Index. All epitopes were showing estimated half-life less than 24hrs. expect VFTEGNIAAMIVNKT which was showing 100hrs (Table 4).

## 4. Discussion

Immunization is one of the major, successful and cost-effective preventive strategy for community health to combat against the fatal infectious diseases globally (Chabot et al., 2004). Although, there is continuous development in the area of vaccines, classical vaccination like whole pathogen immunization is still popular. These types of immunizations are known to produce long lasting and strong immunity, but the major concern is that it may induce strong allergic reactions (Skwarczynski and Toth, 2016). So, peptide-based vaccines (PBVs) or multi epitope vaccines have now become a better choice for safe vaccination. PBVs are a striking alternative approach that depends on selection and usage of short peptide fragments to engineer the stimulation of extremely targeted immuno-protective responses, avoiding allergenic sequences (Li et al., 2014). In this context multi epitope-based vaccines are competent of stimulating strong immunogenic responses and safer option than whole protein-based vaccines. Earlier various studies in public domain are also encouraging the efficacy and impact of multiple epitopes based *in silico* vaccinology (Chaitra et al., 2005; Parida et al., 2007; Wiwanitkit, 2007; Gupta et al., 2010; Shey et al., 2019).

As we all are aware of antimicrobial resistance, which is a severe problem of healthcare at present and affecting millions of people around the globe. Antiviral resistance on the other hand, has been considered as a lesser threat than antibiotic resistance because unlikely drugs, vaccines are used for the prophylactic roles (Kennedy and Read, 2017). However, lately vaccine resistance is also becoming an important and inflammable problem.

Viruses are known for the high mutational rate in very short replication time, which is led by the nucleotide sequence context on the template molecule as well as by external environmental factors. This kind of genetic variation is the guarantee of virus survival in extreme conditions, as the significance of high rate mutation escort to formation of quasi-species or new viral strains (Lauring and Andino, 2010). Single-stranded RNA virus like influenza and marburg often carries error prone polymerases, which habitually induce at least one (range 0.1–10) incorrect base selection during every round of replication and initiate rapid materialization for vaccine resistant (Domingo and Holland, 1997). In this article authors pinpoint three nodes of vaccine that are identification of non-mutagenic, highly antigenic and non-toxic peptides from *Env* gylcoprotein of marburg virus (MRV), which may have potential to point at a direction in designing of a new vaccine to combat marburg virus induced infections.

**Fig. 5.**
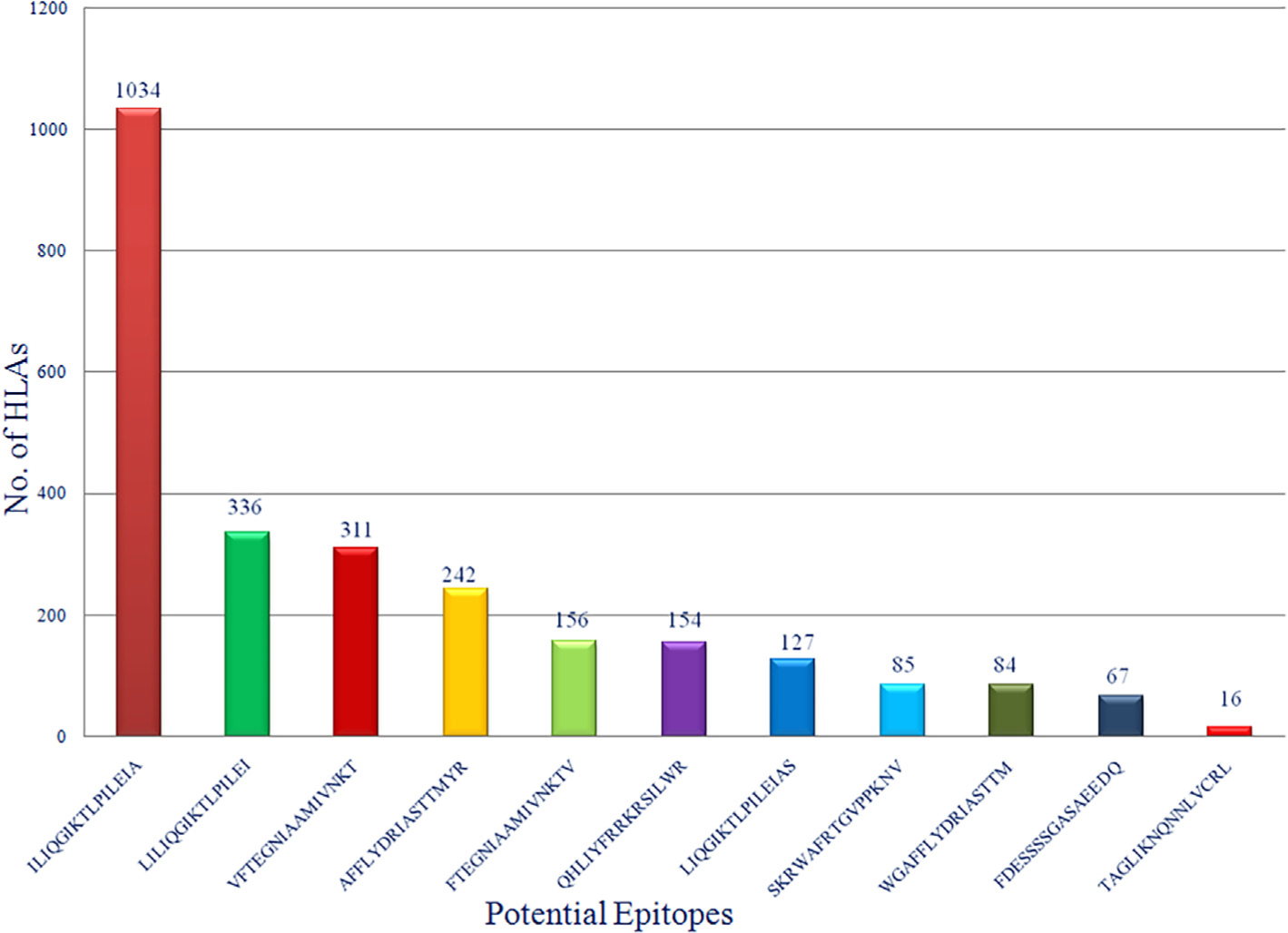
Bar-diagram representation of final 11 epitopes with their HLAs coverage analysis.

*Env* gylcoprotein of any virus is supposed to be a probable target for the vaccine construction, because only *Env* gylcoprotein is accountable for the docking and connection of virus with any kind of human protein or receptor which lead the entry of virus into the cell (Janahi et al., 2017). In current study, authors focused on selection of non-mutagenic, immunogenic and non-toxic epitopes of *Env* gylcoprotein of MRV. As we know, viruses are known for their characteristics of high genomic mutation rates which provides the protection coverage for the virus and this is one of the major causes of vaccine failure (Laughlin et al., 2015). Considering this problem, we have focused on 37 variable strains of *Env* glycoproteins with 89–90% homology. Selected amino acid sequences were considered as input data for Protein Variability Server (PVS) to identify the most probable hot spots of mutations in *Env* glycoprotein. After implementation of PVS techniques, 10 different non variable/mutagenic and antigenic fragments of *Env* glycoprotein were generated. Out of 10, 8 fragments were selected for further screening, those have at least equal to or more than 15 peptides length. In this study authors have only focused on the selection and identification of CD4 + T cell mediated immunity because, CD4 + T-cells activation is an initial and mandatory factor for a competent humoral immune response for the induction of immunoglobulin-G and memory B-cells. CD4 + T-cells primarily recognized by antigen peptides by CD4 co-receptor and only recognized by the major histocompatibility complex (MHC) II protein on antigen-presenting cells; then memory B cells construct a repository of infected virus for the further prophylaxis arrangement (Clem, 2011). Cytotoxic T-cells or TCD8 + have different roles in immunity, which is related to therapeutic understanding not a prophylaxis segment. That’s why for rapid and cost-effective development for prophylaxis vaccine development CD4 + T cell alone is capable and important to induce protective response. NetMHCPanII server was used for the identification of predicted putative CD4 + T-cell epitopic core sequences in each of fragments of *Env* proteins along with their respective binding HLAs. Only CD4 + T-cell epitopes were chosen because CD4 + T-cells are the only immune cells that initially identify antigenic proteins and forms major histocompatibility complex (MHC) II, followed by setting up the configuration of memory B cells for the further prophylaxis arrangement (Gupta et al., 2010). Total 5224 HLAs were listed in the NetMHCpan server (DQ: 2912, DP: 2247, DRB4: 06, DRB1: 15, DRB3: 29, DRB5:15). All eight fragments were screened with all 5224 HLAs. After generation of this huge data, we chose only strong peptide binder among all 8 sets of fragments with lowest affinity score. Best 29, strong binder epitopes were extracted, as mentioned in Table 2. Interferon -gamma (IFN-gamma) potential was also evaluated for all 11 epitopes, and we found in category 1 (IFN-gamma vs Non IFN-gamma) 5 out 11 epitopes were showing potential to induce the IFN-gamma response against viral infection and among 5 epitopes our best top two selected epitopes (ILIQGIKTLPILEIA: 1034 HLAs and LILIQGIKTLPILEI: 336 HLAs) were present those having highest HLA coverages. Second category (IFN-gamma vs other cytokinin) were showed all 11 epitopes were showing better potential to induce the IFN-gamma as compared to other cytokines (details as mentioned in Table 3). About the physiochemical characteristics we found that most of finally selected epitopes have broad range of HLA coverage, but they were not showing good solubility in water and thermodynamically unstable in biological system, since its very usual with all small peptides and nucleosides, they are very prone to degradation via circulating proteases and nucleases. These enzymes are abundantly found in the biological systems, that’s why in this case authors will suggest the nanoparticle (NP)-formulation coting of peptides to shield themselves from proteases enzymes. NPs have the ability to transport weak antigens or vaccines to the mature DCs within the secondary lymph organs. Nano formulation is able to protect peptides, from degradation by proteases. By using NPs as a delivery system, we can initiate stronger immune responses. Once NPs reaches DCs, then controlled release of epitopes can be achieved through chemical modification on their surface, thus activation of DCs can be achieved more efficiently (Jia et al., 2018). Based on all the previously mentioned information we strongly believe that, nano formation system can prove to be an effective and potentiating delivery system for multi-epitopes. Even recently developed COVID-19 vaccine by Pfizer–BioNTech and Moderna also encapsulating the mRNA in lipid nanoparticles (Chaudhary et al., 2021) to save them from nucleases enzymes in biological system. Biodegradable nanoparticles generally made up of poly (D,L-lactic acid-co-glycolic acid)/PLGA are approved for the use of human (Elmowafy et al., 2019). PLGA based peptide NPs are very popular and efficient option of vaccine delivery system for targeting DCs and the development of DCs based cellular vaccines (Athanasiou et al., 2017). So, in that case authors will suggest PLGA nano-formulation will be most suitable candidate for this encapsulation while it already approved by FDA in drug formulations and able to protect the non-water soluble and thermodynamically unstable epitopes. Furthermore, all 29 epitopes were screened based on antigenicity and final 11 epitopes were selected as probable multi epitopes vaccine candidates, with non-toxic properties and high world population coverage (as shown in Fig. 5). This result shows that the proposed epitopes would be significant vaccine contenders for large proportion of the human population which is around 96.78% globally. In short, this study generously focused on a strong prophylactic intervention against MRV with very low possibility of resistance, highly antigenic, non-toxic and high world population coverage. However, the T-cell stimulation potential of the predicted putative CD4 + T-cell epitopes are required to be validated by wet lab experiments for their efficient use as peptide vaccine candidates against marburg virus (MRV).

## 5. Conclusion

In this study, immuno-informatics tools were employed to design a putative vaccine peptide coding for multiple T-cell CD4 + epitopes. Total 11 peptides were minutely screened based on high antigenicity, non-mutagenic, non-toxic and broad HLA coverage. This computer-based study uses strong technical and logical methodologies that help this study to become more precise and reproducible in real time models. The authors are very hopeful, however, the T-cell stimulation potential of these predicted peptides containing the core amino acid sequences are to be validated by using *in vitro* and *in vivo* experiments for their competent use as multiepitope vaccine candidates against MRV infection. This study can be highly useful for designing newer vaccine strategies to prevent and/or lower the death toll attributed to MRV infection in future.

## Supplementary Material

1

## Figures and Tables

**Table 1:** Protein variable fragments of EnvGP of MRV obtained via Protein Variability Server run.

Set No.	Start	End	Sequence	Vaxijen Score (0.4 Threshold)
**1**	3	27	TTCFFISLILIQGIKTLPILEIASN	0.6525(Probable ANTIGEN)
**2**	29	230	QPQNVDSVCSGTLQKTEDVHLMGFTLSGQKVADSPLEASK RWAFRTGVPPKNVEYTEGEEAK TCYNSVTDPSGKSLLLDPPTNVRDYPK CKTIHHIQGQNPHAQGIALHLWGAFFLY DRIASTTMYRGKVFTEGNIAAMIVNKTVHKMIFSRQGQGYRHMNLTSTN KYWTSSNGTQTNDTGCFGTLQEYNSTKNQTCAPSK	0.4850(Probable ANTIGEN).
**3**	240	252	PEIKPTSTPTDAT	1.3071(Probable ANTIGEN).
**4**	254	316	LNTTNPNSDDEDLTTSGSGSGEQEPYTTSDAVTKQGLSST MPPTPSPQPGTPQQGGNNTNHSQ	0.4729(Probable ANTIGEN).
**5**	324	348	NTNTTAQPPMPSHNTTTISTNNTSK	0.4109(Probable ANTIGEN).
**6**	350	354	NLSTLS	0.8725(Probable ANTIGEN).
**7**	365	379	NTQSMATENEKTSAP	0.6994(Probable ANTIGEN)
**8**	381	405	KTTLPPTESPTTEKSTNNTKSPTTM	0.4557(Probable ANTIGEN).
**9**	415	496	SPSSTPNSTTQHLIYFRRKRSIL WREGDMFPFLDGLINAPIDFDPVPNTKTIFDESSSSGASAEEDQHASSNISLTLSYLP	0.6654(Probable ANTIGEN).
**10**	498	680	SENTAYSGENENDCDAELR IWSVQEDDLAAGLSWIPFFGPGIEGLYTAGLIKNQNNL VCRLRRLA NQTAKSLELLL RVTTEERTFSLINRHA IDFLLTRWGGTCKVLGPDCC IGIEDLSRNISEQIDQIKKDEQKEGTG WGLGGKWWTSDWGVLTNLGILLLLSIAVLIALSCICRIFTKYIG	0.4865(Probable ANTIGEN).

**Table 2:** HLA coverage of epitopes with their locations in *Env* protein fragments along with possible antigenicity and toxicity profiling.

Sr. No.	Epitopes	Position	HLA Coverage	Score of ANTIGENICITY and TOXICITY Profiling
**1**	ILIQGIKTLPILEIA	Set 1 from11 to 25	1034	0.6513(Probable ANTIGEN & NON-TOXIC
**2**	IALHLWGAFFLYDRI	Set2 from 134 to 148	458	−0.0643(Probable NON-ANTIGEN & NON-TOXIC).
**3**	ALHLWGAFFLYDRI	Set2 from 135 to 149	646	−0.1142(Probable NON-ANTIGEN & NON-TOXIC).
**4**	LIQGIKTLPILEIAS	Set 2 from 12 to 26	127	0.5381(Probable ANTIGEN & NON-TOXIC)
**5**	WGAFFLYDRIASTTM	Set 2 from 139 to 153	84	0.5262(Probable ANTIGEN & NON-TOXIc).
**6**	LHLWGAFFLYDRIAS	Set 2 from 136 to 150	147	−0.0445(Probable NON-ANTIGEN & NON-TOXIC).
**7**	AFFLYDRIASTTMYR	Set 2 from 141 to 155	242	0.4031(Probable ANTIGEN & NON-TOXIC)
**8**	GIALHLWGAFFLYDR	Set 2 from 133 to 147	11	0.1535(Probable NON-ANTIGEN & NON-TOXIC).
**9**	HLWGAFFLYDRIAST	Set 2 from 137 to 151	282	0.1553(Probable NON-ANTIGEN & NON-TOXIc)
**10**	GAFFLYDRIASTTMY	Set 2 from 140 to 154	113	0.3971(Probable NON-ANTIGEN & NON-TOXIc).
**11**	LILIQGIKTLPILEI	Set 1 from10 to 24	336	0.6611(Probable ANTIGEN & NON-TOXIC).
**12**	QHLIYFRRKRSILWR	Set 9 from 425 to 439	154	1.2274(Probable ANTIGEN & NON-TOXIC)
**13**	IAAMIVNKTVHKMIF	Set 2 from 164 to 178	14	0.0057(Probable NON-ANTIGEN & NON-TOXIC)
**14**	GKSLLLDPPTNVRDY	Set 2 from 101 to 115	58	0.0791(Probable NON-ANTIGEN & NON-TOXIc).
**15**	SLILIQGIKTLPILE	Set 1 from 9 to 23	5	0.6252(Probable ANTIGEN & NON-TOXIC).
**16**	TAGLIKNQNNLVCRL	Set 10 from 545 to 559	16	0.7029(Probable ANTIGEN & NON-TOXIc).
**17**	ERTFSLINRHAIDFL	Set 10 from 580 to 594	1	0.9379(Probable ANTIGEN & NON-TOXIc).
**18**	SKRWAFRTGVPPKNV	Set 2 from 67 to 81	85	0.6970(Probable ANTIGEN & NON-TOXIc).
**19**	GNIAAMIVNKTVHKM	Set 2 from 163 to 177	1	0.2493(Probable NON-ANTIGEN & NON-TOXIC).
**20**	ISLILIQGIKTLPIL	Set 1 from 8 to 22	2	0.7761(Probable ANTIGEN & NON-TOXIC).
**21**	GKVFTEGNIAAMIVN	Set 2 from 156 to 170	142	0.0750(Probable NON-ANTIGEN & NON-TOXIC).
**22**	HLWGAFFLYDRIAST	Set 2 from 137 to 151	116	0.3992(Probable NON-ANTIGEN & NON-TOXIC)
**23**	KVFTEGNIAAMIVNK	Set 2 from 157 to 171	62	0.2910(Probable NON-ANTIGEN & NON-TOXIC).
**24**	VFTEGNIAAMIVNKT	Set 2 from 158 to 172	311	0.4674(Probable ANTIGEN & NON-TOXIC).
**25**	EGNIAAMIVNKTVHK	Set 2 from 161 to 175	6	0.2464(Probable NON-ANTIGEN & NON-TOXIC).
**26**	FTEGNIAAMIVNKTV	Set 2 from 159 to 177	156	0.5746(Probable ANTIGEN & NON-TOXIC).
**27**	RGKVFTEGNIAAMIV	Set 2 from 156 to 172	71	0.2351(Probable NON-ANTIGEN & NON-TOXIC).
**28**	FDESSSSGASAEEDQ	Set 9 from 468 to 482	67	0.4303(Probable ANTIGEN & NON-TOXIC).
**29**	MFPFLDGLINAPIDF	Set 9 from 443 to 457	18	0.3241(Probable NON-ANTIGEN & NON-TOXIC).

**Table 3:** IFN-gamma induction potion of finally selected epitopes. (Using http://crdd.osdd.net/raghava/ifnepitope/).

S.No.	Sequence	Method	IFN-gamma versus Non IFN-gamma	Score	Method	IFN-gamma versus other cytokine	Score
1	ILIQGIKTLPILEIA	SVM based	POSITIVE	0.19866307	MERCI	POSITIVE	1
2	LILIQGIKTLPILEI	SVM based	POSITIVE	0.26377164	SVM	POSITIVE	0.58082894
3	VFTEGNIAAMIVNKT	SVM based	NEGATIVE	−0.045341899	MERCI	POSITIVE	1
4	AFFLYDRIASTTMYR	SVM based	POSITIVE	0.18703322	SVM	POSITIVE	0.57522841
5	FTEGNIAAMIVNKTV	SVM based	NEGATIVE	−0.23191814	MERCI	POSITIVE	1
6	QHLIYFRRKRSILWR	SVM based	POSITIVE	0.28610167	MERCI	POSITIVE	17
7	LIQGIKTLPILEIAS	SVM based	POSITIVE	0.15795841	MERCI	POSITIVE	1
8	SKRWAFRTGVPPKNV	SVM based	NEGATIVE	−0.060456685	MERCI	POSITIVE	1
9	WGAFFLYDRIASTTM	SVM based	NEGATIVE	−0.01994069	MERCI	POSITIVE	1
10	FDESSSSGASAEEDQ	SVM based	NEGATIVE	−0.20096112	SVM	POSITIVE	0.40317329
11	TAGLIKNQNNLVCRL	SVM based	NEGATIVE	−0.42184921	MERCI	POSITIVE	2

**Table 4:** Physiochemical properties of finally selected epitopes. (Using https://pepcalc.com/; https://www.biosyn.com/peptidepropertycalculator/peptidepropertycalculator.aspx and https://web.expasy.org/protparam/).

S. No.	Sequence	Sequence Composition (In percentage)	Mol. Wt.	Estimated solubility & Instability index	Protein-binding Potential (Boman index)	Estimated half-life (Model: mammalian reticulocytes, *in vitro*).
1	ILIQGIKTLPILEIA	Acidic: 6.67 Basic: 6.67 Neutral: 26.67 Hydrophobic: 60	1635.18 g/mol	Poor water solubility, unstable	−1.44 kcal/mol	20 h
2	LILIQGIKTLPILEI	Acidic: 6.67 Basic: 6.67 Neutral: 26.67 Hydrophobic: 60	1677.27 g/mol	Poor water solubility, unstable	−1.65 kcal/mol	5.5 h
3	VFTEGNIAAMIVNKT	Acidic: 6.67 Basic: 6.67 Neutral: 33.33 Hydrophobic: 53.33	1607.97 g/mol	Poor water solubility, unstable	0.19 kcal/mol	100 h
4	AFFLYDRIASTTMYR	Acidic: 6.67 Basic: 13.33 Neutral: 20 Hydrophobic: 60	1855.2 g/mol	Poor water solubility, Stable	1.7 kcal/mol	4.4 h
5	FTEGNIAAMIVNKTV	Acidic: 6.67 Basic: 13.33 Neutral: 20 Hydrophobic: 60	1607.87 g/mol	Poor water solubility, unstable	0.19 kcal/mol	1.1 h
6	QHLIYFRRKRSILWR	Acidic: 0 Basic: 40 Neutral: 13.33 Hydrophobic: 46.67	2072.46 g/mol	Good water solubility, unstable	3.59 kcal/mol	0.8 h
7	LIQGIKTLPILEIAS	Acidic: 6.67 Basic: 6.67 Neutral: 33.33 Hydrophobic: 53.33	1609.09 g/mol	Poor water solubility, stable	−0.88 kcal/mol	5.5 h
8	SKRWAFRTGVPPKNV	Acidic: 0 Basic: 26.67 Neutral: 40 Hydrophobic: 33.33	1743.11 g/mol	Good water solubility, unstable	2.49 kcal/mol	1.9 h
9	WGAFFLYDRIASTTM	Acidic: 6.67 Basic: 6.67 Neutral: 26.67 Hydrophobic: 60	1779.11 g/mol	Poor water solubility, stable	0.48 kcal/mol	2.8 h
10	FDESSSSGASAEEDQ	Acidic: 33.33 Basic: 0 Neutral: 46.67 Hydrophobic: 20	1545.49 g/mol	Good water solubility, unstable	3.52 kcal/mol	1.1 h
11	TAGLIKNQNNLVCRL	Acidic: 0 Basic: 13 Neutral: 40 Hydrophobic: 46.67	1657.06 g/mol	Poor water solubility, stable	1.38 kcal/mol	7.2 h

## Abbreviations:

MRV: Marburg virus
MHF: Marburg Hemorrhagic Fever
WHO: World Health Organization
NIH: National Institutes of Health
CDC: Centre of Disease Control and Prevention
MHC: Major Histocompatibility Complex
*Env* GPs: Envelope Glycoproteins
HLA: Human Leukocyte Antigen
